# Lung microbiota analysis in early-stage lung adenocarcinoma

**DOI:** 10.1128/spectrum.01987-25

**Published:** 2026-03-30

**Authors:** Ke Sun, Bowen Li, Chengye Zhang, Kaichen Zhou, Zhibo Zheng, Chao Gao, Yuxiao Lin, Nan Zhang, Zhina Wang, Ji Li, Lan Song, Naixin Liang, Zhihua Liu

**Affiliations:** 1 School of Basic Medical Sciences, Institute for Immunology, Tsinghua University 731749, Beijing, China; 2 Department of Thoracic Surgery, Peking Union Medical College Hospital, Chinese Academy of Medical Sciences and Peking Union Medical College, Beijing, China; 3 Beijing Institute of Genomics, Chinese Academy of Sciences, China National Center for Bioinformation 74696 , Beijing, China; 4 University of Chinese Academy of Sciences 74519, Beijing, China; 5 Department of Pulmonary and Critical Care Medicine 2, Emergency General Hospital 159795 , Beijing, China; 6 Department of Pathology, Peking Union Medical College Hospital, Chinese Academy of Medical Sciences and Peking Union Medical College, Beijing, China; 7 Department of Radiology, Peking Union Medical College Hospital, Chinese Academy of Medical Sciences and Peking Union Medical College, Beijing, China; 8 Tsinghua-Peking Center for Life Sciences, Tsinghua University 12442, Beijing, China; 9 State Key Laboratory of Membrane Biology 553740, Beijing, China; Nova Southeastern University, Fort Lauderdale, Florida, USA

**Keywords:** lung microbiota, early-stage lung adenocarcinoma, 16S rRNA, microbiota

## Abstract

Lung adenocarcinoma (LUAD) is a predominant subtype of lung cancer and is highly lethal. The lung tissue microbiota has been implicated in lung cancer biology but remains incompletely defined at early stages. We enrolled 40 patients with early-stage LUAD. After sequencing quality control, 34 early-stage LUAD tissues and 17 distal noncancerous lung (NC) tissues were retained, including 14 matched LUAD-NC pairs. Microbiota differences were profiled by 16S rRNA sequencing. Early-stage LUAD tissues contained more low-prevalence taxa, with higher richness metrics, but evenness-sensitive indices remained unchanged. β-Diversity analyses further supported that differences were driven primarily by the presence and absence of community membership rather than shifts in high-abundance taxa. Differential abundance analysis using MaAsLin2 identified a set of genera associated with early-stage LUAD, including *unclassified_f__Oscillospiraceae* and *norank_c__Eremiobacteria*, as well as several genera decreased in tumors. The microbial features identified through stability screening moderately distinguished early-stage LUAD from NC tissues in out-of-bag validation. Network analysis revealed denser and more clustered topology in early-stage LUAD along with a shift in central taxa compared with NC. Finally, inferences based on PICRUSt2 suggested redistribution of predicted functional potentials across modules linked to microbial cell-envelope programs, ecological interaction traits, and immunometabolic pathways. These findings collectively reveal microbiota alterations in lung tissues associated with early-stage LUAD and support the feasibility of early detection based on microbiota characteristics.

**IMPORTANCE:**

Microbial dysbiosis has been recognized as a critical factor in the development of lung cancer, but most current research has focused on the gut rather than the lung microbiota. Identifying the key microbes involved in the development of lung adenocarcinoma is therefore essential. In this study, we analyzed the composition and structure of the microbiota in early-stage lung adenocarcinoma tissues in comparison to noncancerous lung tissues. Our findings provide novel insights into the diagnostic potential of the lung microbiota in early-stage lung adenocarcinoma and enhance our understanding of its involvement in tumor progression and the discovery of potential therapeutic targets.

## INTRODUCTION

Lung cancer is one of the most prevalent malignancies worldwide, associated with high morbidity and mortality rates. Over 2.2 million people are diagnosed with lung cancer annually (1), imposing a substantial burden on society. Among lung cancer patients, 40% to 50% receive a diagnosis of LUAD (2), which is the most common type of non-small cell lung cancer (NSCLC). In clinical practice, initial evaluation relies primarily on imaging modalities, such as chest radiography, positron emission tomography (PET), computed tomography (CT), and magnetic resonance imaging (MRI) (3). Although indispensable for detection and staging, imaging alone has limited specificity for subtype classification and early lesion discrimination, and definitive diagnosis still depends on pathological assessment. Accordingly, complementary biomarkers that improve early-stage risk stratification and support subtype-specific diagnosis remain of interest, including microbial signals detected in respiratory specimens (4, 5).

The lung was long considered sterile, but recent studies have established that the lower respiratory tract harbors low-biomass microbial communities that vary with local environmental conditions (6–10). Alterations in the lung microbiota have been proposed to influence inflammation and disease progression (11–14). The enrichment of *Streptococcus* and *Veillonella* in the lower airways has been associated with activation of ERK and PI3K signaling pathways, and interference of commensal bacteria can modulate tumor progression in mouse models (14, 15). These observations support the concept that characterizing lung microbiota alterations may provide insights into lung disease biology and inform biomarker development.

Recent work has begun to profile early-stage LUAD microbiota using diverse sampling strategies and analytical platforms (16–21). An integrative study combining ultra-deep metagenomics with host transcriptomics and proteomics in non-smokers with early-stage LUAD reported multi-kingdom microbial signals and host-microbe correlations and proposed a six-bacterial-marker model for disease discrimination (21). In parallel, a biopsy-based profiling study has suggested that a limited set of tumor-associated genera can support classifier construction with high performance, highlighting the diagnostic potential of tissue microbial features (17). However, cross-study concordance of specific taxa remains a challenge, in part because microbial profiles are sensitive to sampling sites and to the methodological challenges of low-biomass tissue microbiota analysis (20).

In this study, early-stage LUAD tissues and partially matched noncancerous lung tissues were profiled by 16S rRNA sequencing, followed by contamination preprocessing and mixed-effects modeling to account for patient-level pairing. In addition to identifying early-stage LUAD-associated taxa, a patient-level cluster-bootstrap stability selection framework was applied to derive a compact set of robust genus-level markers and to estimate out-of-bag performance. Correlation network analysis and functional inference using PICRUSt2 were further used to detect community restructuring and predicted functional remodeling in early-stage LUAD. Together, these analyses support the potential utility of lung tissue microbiota features as biomarkers for early-stage LUAD.

## MATERIALS AND METHODS

### Study design and samples

A total of 40 participants from Peking Union Medical College Hospital were recruited from June 2021 to June 2023. All patients were diagnosed with LUAD. Baseline clinical information included age, gender, tumor stage, smoking history, and other disease history (Table 1).

**TABLE 1:** Clinical baseline characteristics of patients in this cohort^*a*^

Characteristics	All	Patients with retained tumor samples	Patients with retained noncancerous samples
*N*	40	34	17
Age (mean ± SD)	62.2 ± 10.5	62.9 ± 10.0	60.9 ± 10.4
Male/female (No.)	9/31	6/28	3/14
TNM tumor stage, *n* (%)			
IA1	3 (7.5)	2 (5.9)	3 (17.6)
IA2	10 (25.0)	6 (17.6)	1 (5.9)
IA3	15 (37.5)	14 (41.2)	5 (29.4)
IB	8 (20.0)	8 (23.5)	8 (47.1)
IIA	2 (5.0)	2 (5.9)	–^*b*^
IIB	2 (5.0)	2 (5.9)	–^*b*^
Smoking status, *n* (%)			
Smoker	10 (25.0)	7 (20.6)	3 (17.6)
Non-smoker	30 (75.0)	27 (79.4)	14 (82.4)
Suffering from other diseases			
Hypertension (yes), *n*	13 (32.5)	11 (32.4)	6 (35.3)
Diabetes (yes), *n*	8 (20.0)	7 (20.6)	5 (29.4)

^*a*^ Fourteen patients contributed paired lung adenocarcinoma and noncancerous lung samples. Columns represent overlapping patient subsets.

^*b*^ “–” indicates that the corresponding category was not observed in that subgroup and therefore no value is reported.

Inclusion criteria included subjects undergoing routine surgery at Peking Union Medical College Hospital who had not received antibiotic or neoadjuvant therapy within 2 months before surgery, were aged 18 to 80 years, had a pathological diagnosis of LUAD with a single tumor, had no history of other cancers, and had no other lung diseases.

### Sample collection

The LUAD tissues were collected during surgery. Immediately after the surgeon removed the lesion, the tissues were placed in liquid nitrogen for long-term storage. The lung cancer tissues were sectioned and further examined by the pathologist to ensure accurate lesion sampling. Additionally, distal noncancerous lung tissues from the same patients were collected at the same time. NC tissues were collected more than 5 cm away from the cancer lesion to avoid potential localized effects of the cancer. All devices, surgical instruments, and tissue collection tubes that came into contact with lung tissues during the process of sample collection were sterile.

### DNA extraction

To minimize environmental contamination, DNA extraction was conducted in a sterile and negative pressure isolator. The collected tissues were weighed and homogenized in lysis solution (10 mM Tris-HCl [pH 8.0], 0.1 M EDTA [pH 8.0], 0.5% SDS) with proteinase K (0.2 mg/mL). Then, DNA was extracted from the tissues using the DNeasy PowerSoil Kit (Qiagen, Germany). After DNA quantity and quality were determined using the Epoch2 enzyme labeler (BioTek, USA), DNA was subjected to 16S rRNA amplicon sequencing.

### 16S rRNA amplicon sequencing

16S rRNA sequencing and microbial analysis of patient tissues were performed by Majorbio Bio-Pharm Technology Co. Ltd. (Majorbio, China). The bacterial 16S rDNA gene V3–V4 highly variable region was amplified using the following primers (338F: 5′- ACTCCTACGGGGAGGCAGCAG-3′, 806R: 5′-GGACTACHVGGGGTWTCTAAT-3′) (22). The PCR products were purified using the AxyPrep DNA Gel Extraction Kit (Axygen, USA), followed by paired-end sequencing on the Illumina MiSeq PE300 platform (Illumina, USA). The Quantus Fluorometer (Promega, USA) was used to quantify the PCR products. The raw data obtained were demultiplexed and quality filtered by fastp (v0.23.4). The lysis buffer controls, DNA extraction blank reagent in the same batch, and PCR blank controls using DNA extraction blank were used as negative controls to assess background contamination. After quality control, 34 tumor samples and 17 distal noncancerous samples were retained, including 14 matched tumor-NC pairs. In total, microbial 16S rRNA sequencing data were obtained from 34 LUAD tissues and 17 NC tissues.

### Microbiota data analysis

Sequencing reads were quality-filtered using fastp (v0.23.4) and merged using FLASH (v1.2.11). Merged reads were processed in QIIME 2 using the DADA2 plugin with default parameters for denoising, chimera removal, and inference of amplicon sequence variants (ASVs). Taxonomic assignment was performed using the QIIME 2 classify-sklearn (naive Bayes) classifier against the SILVA 138.2 (16S rRNA, Bacteria) reference database with a confidence threshold of 70%. To mitigate potential background contamination, sequences detected in negative controls were modeled and removed using SCRuB (23). Prior to downstream analyses, ASVs annotated as chloroplast or mitochondrial sequences were excluded, and low-prevalence features were filtered by retaining ASVs detected in at least two samples with a total abundance of ≥10 reads across the data set. Per-sample read depth distributions and read retention across filtering, denoising, and chimera-removal steps (including negative controls) are summarized in Table S1.

α-Diversity indices (Sobs, ACE, Chao1, Shannon, and Simpson) were calculated at the genus and ASV levels using data sets rarefied to the minimum sequencing depth (3,439 reads per sample). Differences between early-stage LUAD and NC tissues were tested using linear mixed-effects models (LMM) with tissue type as a fixed effect and patient ID as a random intercept. Multiple testing across indices was controlled using the Benjamini-Hochberg procedure, and adjusted *P* values are reported as FDR. β-Diversity was assessed using Bray-Curtis, binary-Hamming, unweighted UniFrac, and weighted UniFrac distances. Principal coordinate analysis (PCoA) was used for ordination. Differences in community composition were tested by PERMANOVA (Adonis).

Differential taxa were identified using MaAsLin2 with the compound Poisson linear model (CPLM). Before modeling, genus-level features were filtered using a prevalence threshold of 15%. Tissue type was included as a fixed effect and patient ID as a random effect. Multiple testing was controlled using Benjamini-Hochberg correction. Adjusted *P* values were presented as *q* values, and taxa with *q* < 0.05 were considered significantly different. For LEfSe analysis, CPM-normalized abundances were used, and Kruskal-Wallis *P* < 0.05 with an LDA cutoff >3.5 was used to identify differential taxa.

Marker selection was conducted using a patient-level cluster bootstrap stability selection framework combined with an elastic net logistic regression model. Resampling was performed at the patient level to prevent information leakage between paired samples. Features were CLR-transformed after adding a pseudocount of 1 and subsequently Z-score standardized. The elastic net model was fitted during each bootstrap iteration. Classifiers were ranked by selection frequency, with stable selection markers defined as those exceeding a preset frequency threshold of 60%. Predictive performance was estimated from aggregated out-of-bag sample-level predictions and summarized using ROC-based metrics.

Correlation networks were inferred using Spearman correlations on the genus level. Edges were retained when |ρ| ≥ 0.7 and *P* < 0.05 (two-sided). Network topology was summarized using density and clustering coefficient. Node importance was quantified using degree centrality (DC), closeness centrality (CC), and betweenness centrality (BC). Robust key nodes were defined based on patient-level bootstrap stability and centrality ranking.

Functional potential was inferred from 16S profiles using PICRUSt2 to obtain predicted Level 3 pathway and KEGG Orthology (KO) abundances. Level 3 pathway abundances were normalized and CLR-transformed after adding a small pseudocount, and differences between tissue types were tested using LMM with patient ID as a random intercept. Multiple testing was controlled by the Benjamini-Hochberg procedure. For KO-level analysis, KO sets linked to host-microbiota interaction processes relevant to lung cancer were curated from the literature (LPS/lipid A, peptidoglycan, secretion systems, biofilm/quorum sensing (QS), fatty-acid metabolism, beta-lactam resistance, SCFA metabolism, tryptophan/indole metabolism, redox/stress response, and nitrogen/sulfur metabolism) and grouped into functional modules. KO abundances were normalized and CLR-transformed with a small pseudocount, and KO associations with tissue type were tested using LMM. KO stability was assessed by leave-one-patient-out refitting, summarizing effects by the median LOPO effect estimate (β_median) and sign consistency. KOs were retained using pre-specified criteria including prevalence ≥ 0.20, |β_median| ≥ 0.30, and sign consistency ≥ 0.90. Retained KOs were partitioned into LUAD-upregulated and LUAD-downregulated subsets based on the sign of β_median, and direction-specific module scores were computed and tested using LMM, with Benjamini-Hochberg correction applied within each direction.

### Statistical analysis

Patient age was presented as the mean ± standard deviation (SD), and other characteristics, including gender, tumor node metastasis (TNM) stage, and smoking status, were summarized as counts and percentages. Unless otherwise specified, statistical tests were two-sided. For analyses involving paired sampling, within-patient correlation was accounted for using LMM or patient-level resampling where applicable. PERMANOVA was performed using unrestricted permutations. All microbiota analyses were conducted in R (v4.4.3). Clinical characteristics comparisons were performed in SPSS v24 (SPSS Inc., IL). Graphs were plotted using GraphPad Prism v8.0 and R. A nominal *P* < 0.05 was considered statistically significant where applicable. Multiple-comparison correction was performed using the Benjamini-Hochberg procedure, with adjusted *P* values reported as FDR for LMM-based diversity and functional module analyses and as *q* values for MaAsLin2 differential abundance testing.

## RESULTS

### Clinical characteristics

We recruited 40 patients with unilateral lobar masses from Peking Union Medical College Hospital between 2021 and 2023. All of them were newly diagnosed with early-stage LUAD through pathological examination. The detailed clinical characteristics of all patients, including age, gender, TNM stage, and smoking status, are shown in Table 1. Thirty-one patients were female (77.5%) with an average age of 62.2 years. Most patients were non-smokers (30/40, 75.0%), whereas 10/40 (25.0%) reported a smoking history. None of them had received anticancer treatment, radiation therapy, or antibiotics within the two months prior to the study. A total of 34 tumor tissue samples and 17 noncancerous lung samples from LUAD patients were retained for downstream microbial analysis.

### Microbial profile of early-stage lung adenocarcinoma tissues

**Fig 1:**
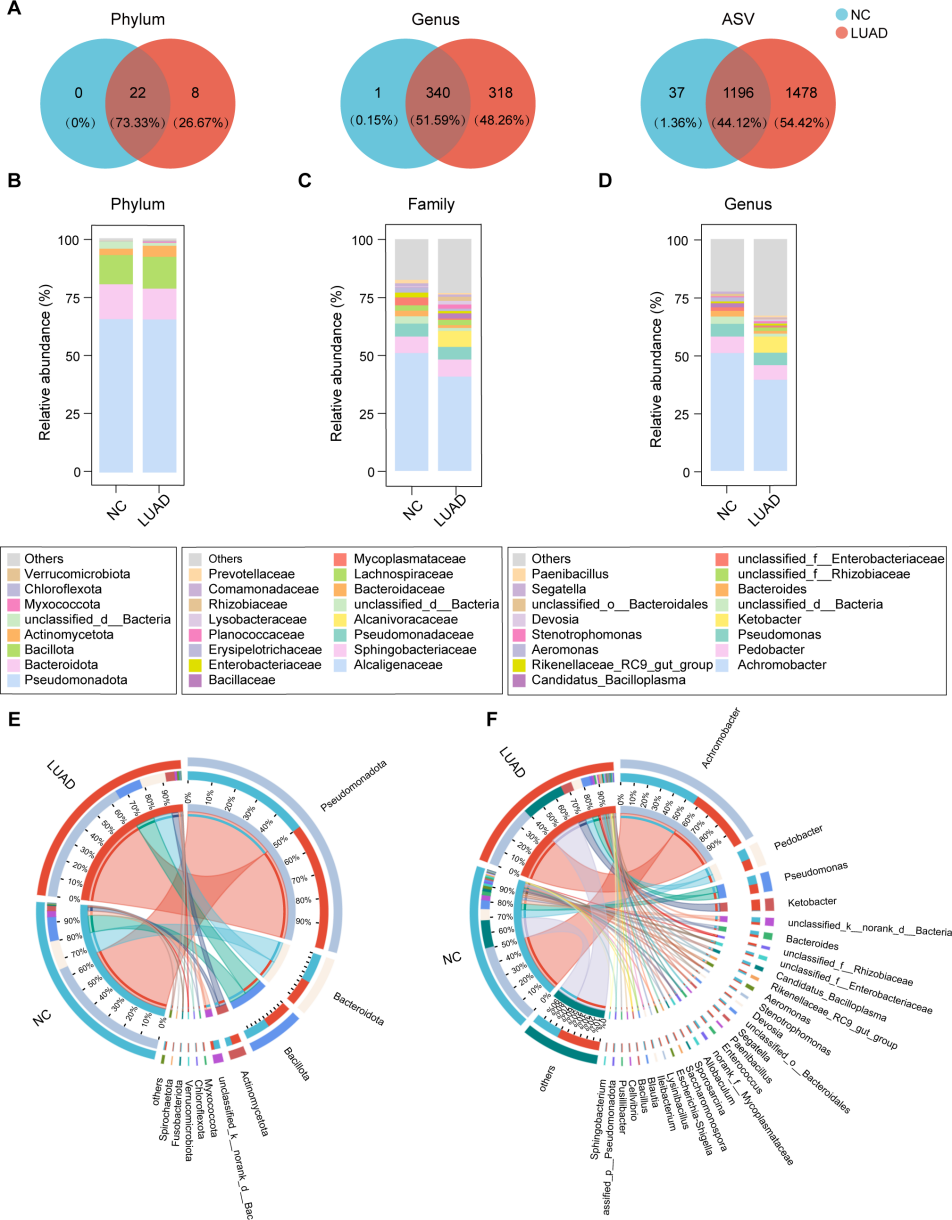
Microbial community composition in NC and early-stage LUAD tissues. (**A**) Venn diagrams showing shared and unique taxa between NC and LUAD groups at the phylum, genus, and ASV levels. (**B–D**) Stacked bar plots showing relative abundances of major taxa at the phylum (**B**), family (**C**), and genus (**D**) levels. (**E and F**) Circos plots summarizing the compositional distribution of the lung microbiota between groups at the phylum (**E**) and genus (**F**) levels.

Sequencing reads from NC tissues and early-stage LUAD tissues were denoised into 2,711 ASVs, which were further taxonomically categorized into 30 phyla and 659 genera. Venn diagrams showed a marked imbalance in shared versus unique features between groups, indicating an expansion of low-prevalence and low-abundance taxa in tumors (Fig. 1A). Then, we characterized the composition of bacterial communities at different taxonomic levels. At the phylum level, *Pseudomonadota*, *Bacteroidota*, *Bacillota,* and *Actinobacteriota* predominated in both NC and early-stage LUAD tissues (Fig. 1B and E). At the family level, early-stage LUAD tissues showed a higher relative contribution from Bacillales-related lineages, including *Bacillaceae*, *Rhizobiaceae*, and *Paenibacillaceae* (Fig. 1C). At the genus level, *Achromobacter* remained the most abundant genus in both groups but showed a lower relative abundance in early-stage LUAD tissues (Fig. 1D). Notably, *Ketobacter* was detectable in early-stage LUAD tissues (mean relative abundance 6.9%) but was rare or absent in NC tissues (Fig. 1D and F). In addition, several LUAD-associated genera, such as *Bacillus* and *Devosia*, were observed at higher relative abundances in early-stage LUAD tissues (Fig. 1D and F). Overall, these results indicate that early-stage LUAD tissues retain a broadly similar high-level taxonomic composition to NC tissues, while exhibiting an increased representation of additional taxa at genus and ASV level.

**Fig 2:**
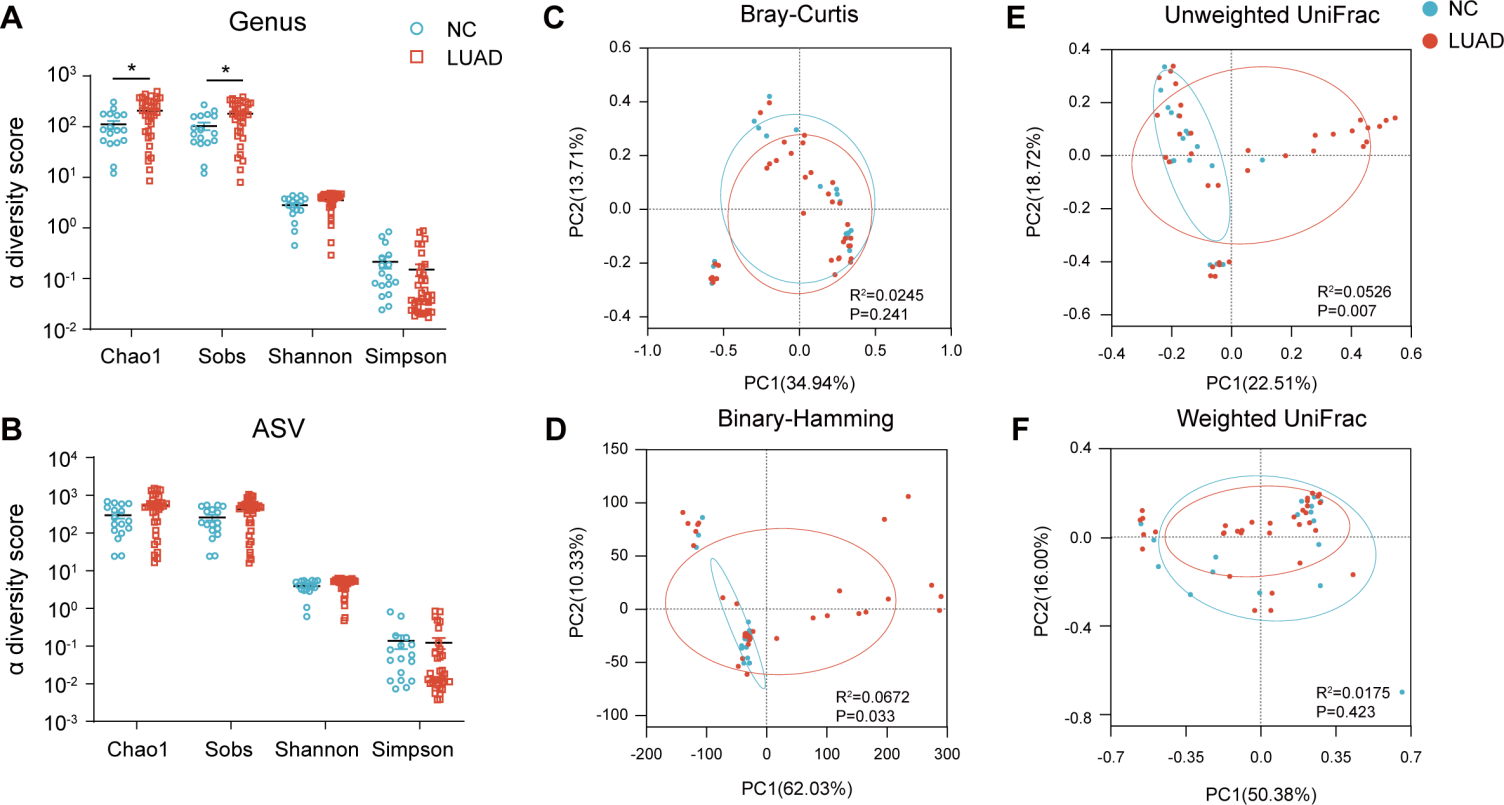
α-Diversity and β-diversity of lung microbiota in NC and early-stage LUAD tissues. (**A and B**) Differences in α-diversity between NC and early-stage LUAD tissues based on the Chao1, Sobs, Shannon, and Simpson at the genus (**A**) and ASV (**B**) levels. Group differences were tested using linear mixed-effects models (LMM). Adjusted *P* values for group comparisons are shown. (**C–F**) Principal coordinate analysis (PCoA) based on Bray-Curtis (**C**), binary-Hamming (**D**), Unweighted UniFrac (**E**), and weighted UniFrac (**F**) distances at the genus level. PERMANOVA (Adonis) results are shown on each ordination plot (*R*² and *P* value). Ellipses indicate group-wise dispersion for each tissue group. *P < 0.05.

Next, we assessed the α-diversity of the microbiota. At the genus level, richness-related indices, including Sobs and Chao1, were significantly higher in early-stage LUAD tissues compared to NC tissues (FDR < 0.05; Fig. 2A). Conversely, Shannon and Simpson indices showed no significant differences between groups, which are sensitive to evenness (FDR > 0.05; Fig. 2A). α-Diversity at the ASV level did not differ significantly between early-stage LUAD tissues and NC tissues (FDR > 0.05; Fig. 2B). Together, these results indicate that LUAD-associated changes are driven primarily by increased taxa richness rather than global shifts in community evenness.

β-Diversity was assessed by PCoA based on Bray-Curtis, binary-Hamming, unweighted UniFrac, and weighted UniFrac distances at the genus level (Fig. 2C through F). Significant separation between early-stage LUAD and NC tissues was observed using binary-Hamming and unweighted UniFrac distances (*P* < 0.05; Fig. 2D and E), both of which emphasize community membership and are more sensitive to low-abundance taxa. In contrast, abundance-weighted metrics did not show significant separation (Fig. 2C and F). Collectively, these findings support the existence of lung microbiota composition alterations driven by the presence or absence of microbial community members in early-stage LUAD tissues, consistent with the expansion of low-prevalence taxa observed at the genus and ASV levels.

### Specific microbiota features in early-stage lung adenocarcinoma tissues

**Fig 3:**
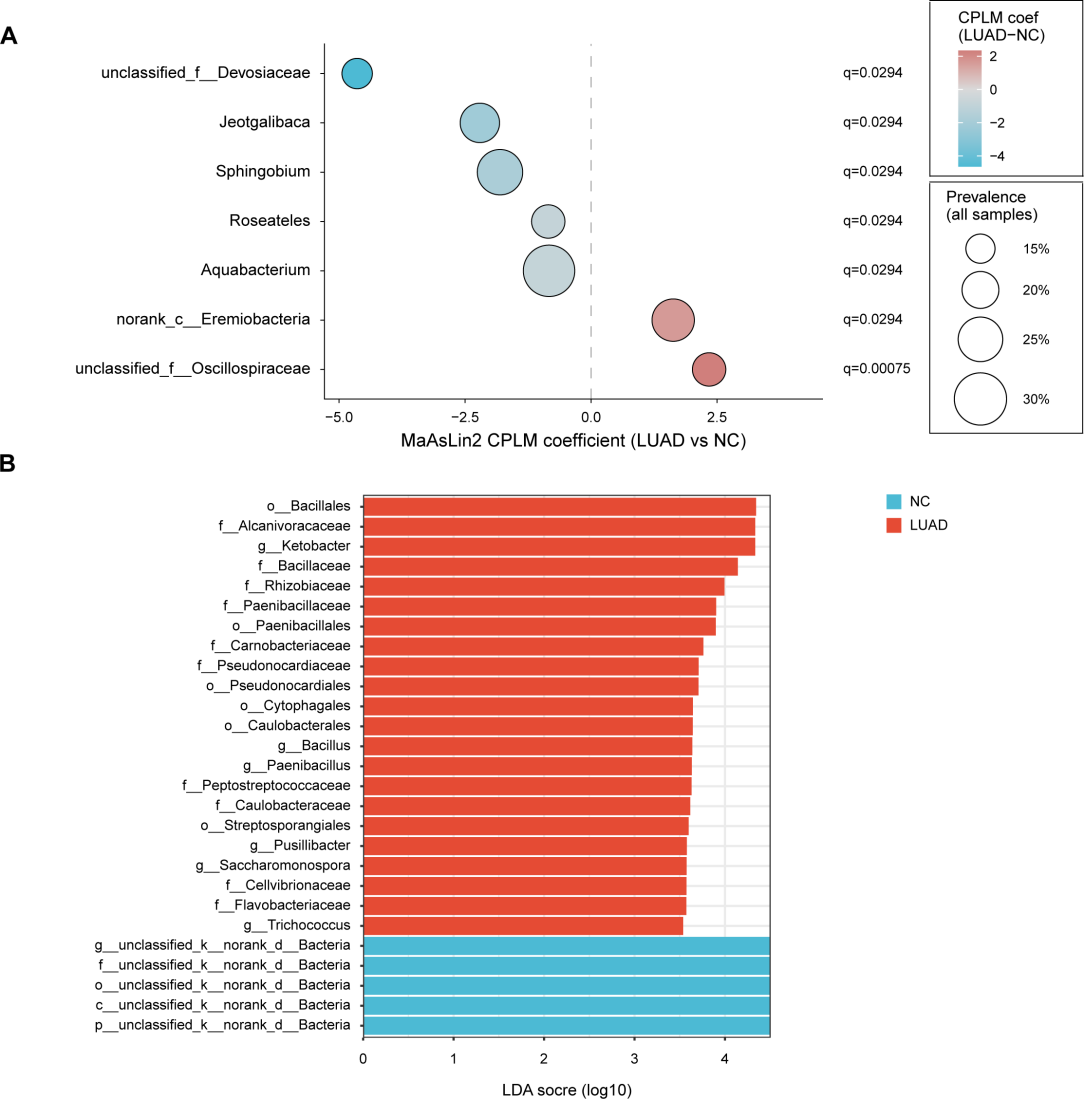
Differential taxa associated with early-stage LUAD identified by paired-design modeling and LEfSe. (**A**) MaAsLin2 results for genus-level differential abundance using the CPLM. Points show model coefficients (LUAD vs NC). Point size denotes prevalence across all samples, and color indicates effect direction. Multiple testing-adjusted *q* values are annotated for significant taxa. (**B**) LEfSe analysis showing discriminant taxa across the taxonomic hierarchy. Bars represent LDA scores (log10), with taxa enriched in LUAD (red) or NC (blue), using the cutoff indicated in the figure (LDA > 3.5; *P* < 0.05).

To identify early-stage LUAD-associated taxa while accounting for the partially paired design, we performed differential abundance testing at the genus level using MaAsLin2. Seven differentially abundant genera were identified between NC and early-stage LUAD tissues (*q* < 0.05; Fig. 3A). *Unclassified_f__Oscillospiraceae* and *norank_c__Eremiobacteria* were significantly enriched in early-stage LUAD tissues (Fig. 3A). In contrast, five genera were significantly depleted in early-stage LUAD tissues relative to NC tissues, including *Aquabacterium*, *Roseateles*, *Sphingobium*, *Jeotgalibaca*, and *unclassified_f__Devosiaceae* (Fig. 3A). Several additional genera showed near-threshold associations (Table S2). Specifically, *unclassified_f__Rhizobiaceae*, *Thermobifida*, *Romboutsia*, *Dubosiella*, *Sellimonas*, and *Bacillus* tended to be higher in early-stage LUAD tissues, whereas *Corynebacterium* tended to be lower. These results indicate that early-stage LUAD is associated with genus-level shifts encompassing both tumor-enriched and tumor-depleted taxa.

As a complementary analysis, LEfSe was used to visualize discriminant taxa across the taxonomic hierarchy (Kruskal-Wallis *P* < 0.05, LDA > 3.5; Fig. 3B). Consistent with the MaAsLin2 results, LEfSe highlighted enrichment of *Bacilli* and *Gammaproteobacteria* at higher ranks and identified several early-stage LUAD-enriched genera, including *Ketobacter*, *Bacillus*, *Paenibacillus*, *Pusillibacter*, *Saccharomonospora*, and *Trichococcus*, supporting a *Bacillales*-linked signal in early-stage LUAD.

### Microbial marker discovery in early-stage lung adenocarcinoma and model discrimination performance

Given that a subset of taxa showed associations with early-stage LUAD in the differential abundance analysis, we subsequently investigated whether these community alterations could be refined into microbial signatures for distinguishing early-stage LUAD tissues from NC tissues. Following a thorough stability screening process, five microbial genus-level markers were identified (selection frequency > 0.60). Among these, *Chryseobacterium* and *Candidatus_Saccharimonas* were the most consistently selected taxa, followed by *Bacillus*. *Staphylococcus* and *Macellibacteroides* were also retained as core members of the signature (Table S3).

**Fig 4:**
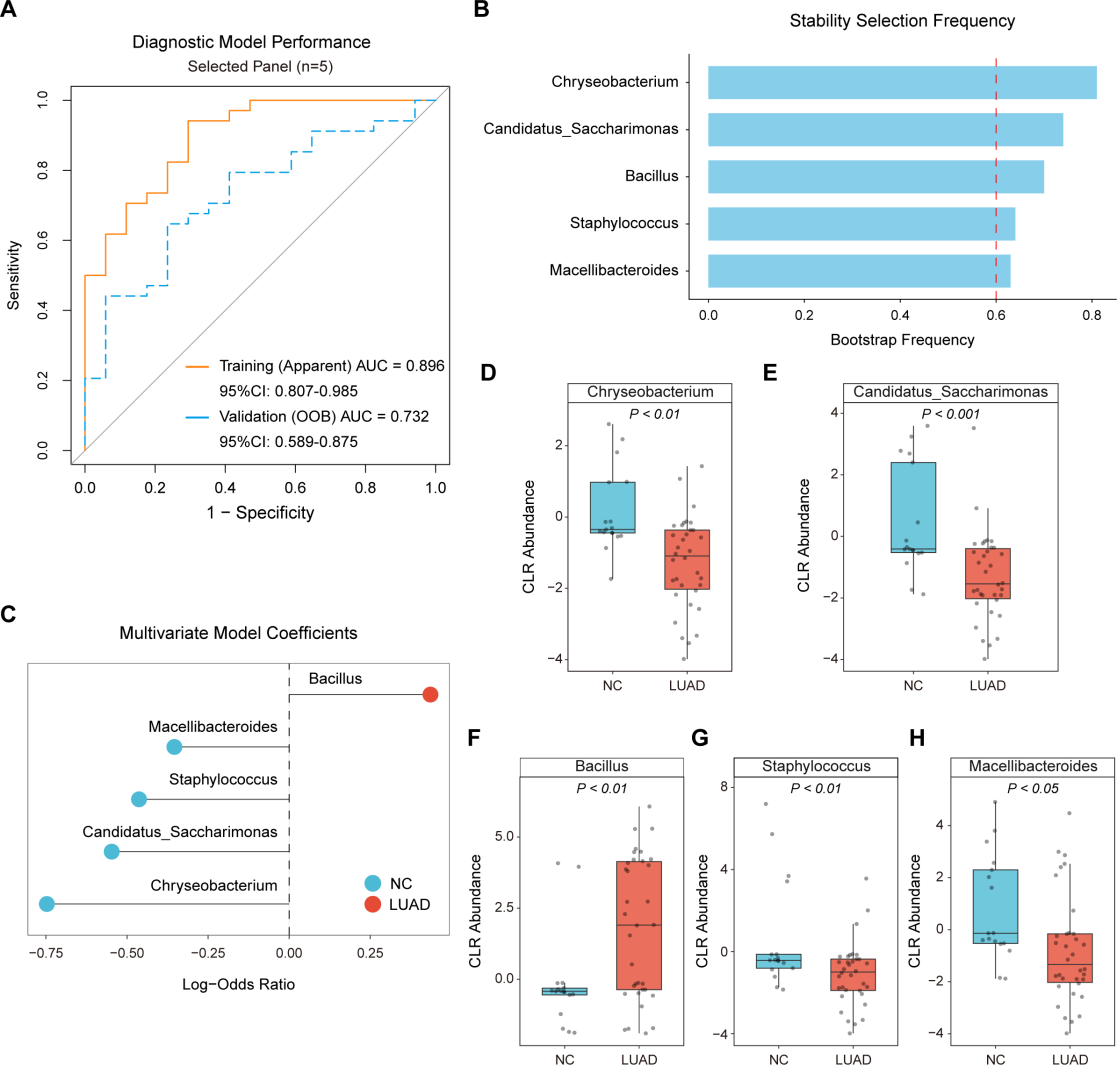
Identification of core genus-level microbial markers and diagnostic model performance. (**A**) ROC curves for the marker panel, showing apparent performance in the training fit and OOB validation performance derived from patient-level resampling. AUC values and 95% confidence intervals (CI) are indicated. (**B**) Stability selection results showing selection frequency of each genus. The dashed vertical line indicates the predefined stability threshold (0.60). (**C**) Multivariable model coefficients (log-odds ratios) for the selected marker panel. (**D–H**) CLR-transformed abundances of each selected genus in NC and LUAD tissues. *P* values for group comparisons are shown above each panel.

A multivariable diagnostic model constructed from these five genera showed strong apparent discrimination in the training fit (AUC = 0.896, 95% CI: 0.807–0.985) (Fig. 4A; Table S4). Performance remained moderate in out-of-bag (OOB) validation, yielding an AUC of 0.732 (95% CI: 0.589–0.875), supporting the potential generalizability of this signature despite the limited sample size. *Bacillus* showed higher relative abundance in early-stage LUAD tissues, whereas *Candidatus_Saccharimonas*, *Macellibacteroides*, *Chryseobacterium*, and *Staphylococcus* were more abundant in NC tissues (Fig. 4B through H). Together, these directionally opposite shifts across the five genera support a composite microbial signature that differentiates early-stage LUAD from noncancerous lung.

### Microbial correlation networks in early-stage lung adenocarcinoma tissues and noncancerous tissues

**Fig 5:**
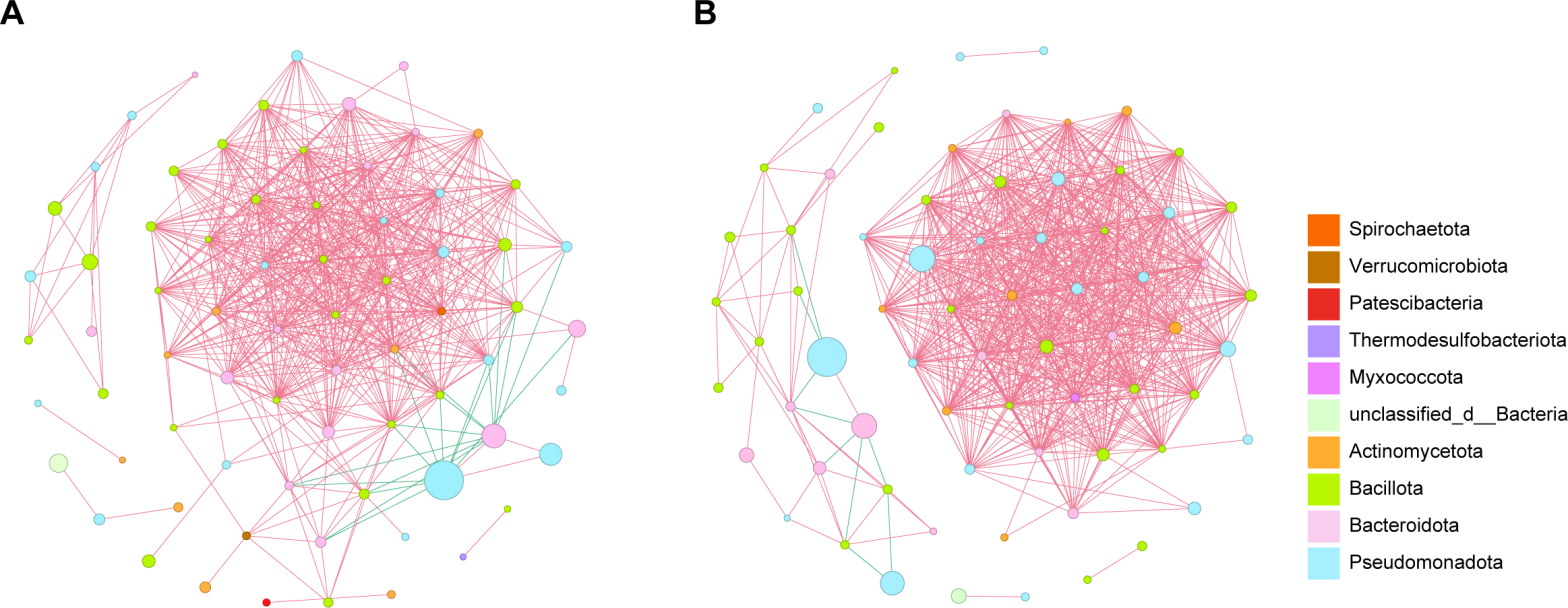
Genus-level correlation network structure in NC and early-stage LUAD tissues. (**A and B**) Spearman correlation networks constructed separately for NC (**A**) and early-stage LUAD (**B**) groups at the genus level. Edges represent significant correlations (|ρ| ≥ 0.7, *P* < 0.05). Nodes are colored by phylum, and the red and green lines represent positive and negative correlations, respectively. Node size is proportional to mean abundance.

To evaluate correlations in the lung microbiota of NC and early-stage LUAD tissues, we constructed the correlation networks of genus taxa using Spearman correlations (Fig. 5A and B). The networks revealed distinct co-occurrence structures between NC and early-stage LUAD tissues. The early-stage LUAD network (mean degree, 21.30; clustering coefficient, 0.83; density, 0.31) showed higher global connectivity and local clustering than the NC network (mean degree, 16.76; clustering coefficient, 0.73; density, 0.24), indicating a denser and more locally clustered co-occurrence structure in tumor tissues. Centrality analyses indicated a shift in influential genera between groups. In the NC network, *Bifidobacterium* ranked highest across centrality measures, with additional highly connected nodes including *Christensenellaceae_R-7_group* and *Faecalibaculum* (Table S5). In contrast, the early-stage LUAD network contained multiple hub-like genera with elevated degree and closeness centrality, including *Paenibacillus*, *Planococcus*, *unclassified_f__Planococcaceae*, *Cellvibrio*, *Arenibacter*, *Albibacterium*, and *norank_f__Sandaracinaceae* (Table S6). Genera with the highest betweenness centrality in early-stage LUAD tissues included *unclassified_o__Bacteroidales*, *Faecalibacterium*, *Carnobacterium*, and *unclassified_f__Rhizobiaceae*, suggesting potential connector roles between subnetworks (Table S6).

To assess robustness, patient-level cluster bootstrap resampling was applied to identify taxa whose centrality patterns were stable to resampling. After applying prevalence and abundance filters, two genera were consistently recovered as bootstrap-stable core nodes in NC tissues, including *Pedobacter* and *Achromobacter* (Table S7). In early-stage LUAD tissues, bootstrap-stable core nodes included *Planococcus* and *unclassified_f__Rhizobiaceae* (Table S7). Collectively, these analyses indicate that early-stage LUAD tissues exhibit a denser and more locally clustered co-occurrence structure than NC tissues and show a distinct set of central and connector taxa, with a subset of genera demonstrating reproducible network centrality under patient-level resampling.

### Functional profile of the microbiota in early-stage lung adenocarcinoma tissues

**Fig 6:**
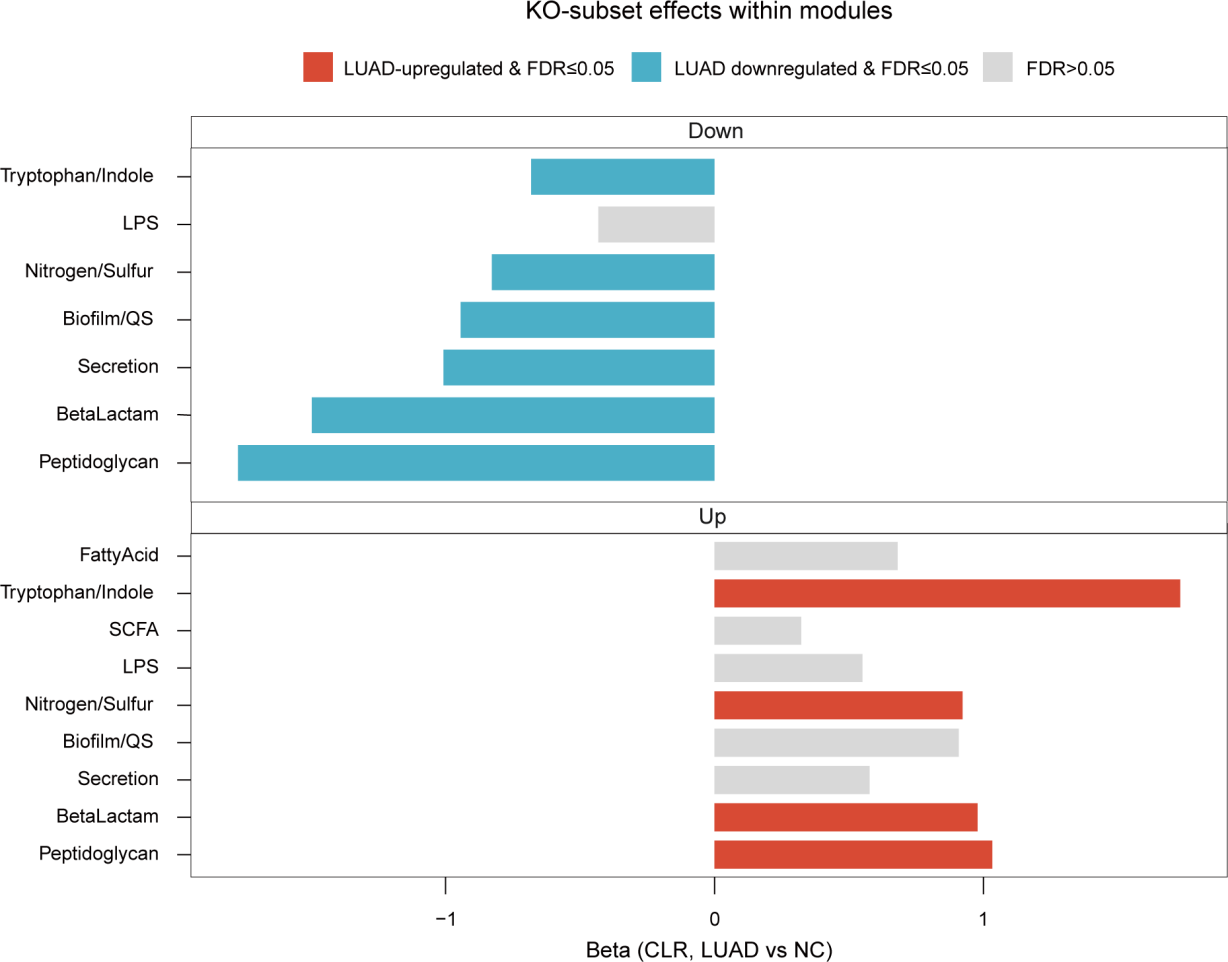
Remodeling PICRUSt2-inferred functional modules in early-stage LUAD tissues. PICRUSt2-predicted KOs were grouped into functional modules. Within each module, KOs were separated into LUAD-upregulated and LUAD-downregulated subsets based on the direction of association, and subset-specific module scores were computed from CLR-transformed KO abundances. Bars show the estimated LUAD-NC effect (β) for each module subset. Modules with FDR < 0.05 are highlighted, and non-significant modules are shown in gray.

To explore the potential functional differences between the lung microbiota of noncancerous and LUAD tissues, we performed KEGG pathway analysis using the PICRUSt2 algorithm. Some KOs were aggregated into functional modules that had been reported in the literature as potentially associated with lung cancer development (24–34). At the Level 3 pathway level, no pathways remained significant after FDR correction (Table S8). In contrast, notable differences in inferred functional potential were observed at the KO/module level. When the KOs were separated into LUAD-upregulated and LUAD-downregulated subsets and summarized at the module level, multiple modules showed significant and directionally consistent alterations (FDR < 0.05). Because LUAD-downregulated and LUAD-upregulated module scores were computed from disjoint KO subsets within each module, opposite-direction changes across subsets indicate redistribution within modules rather than uniform changes across pathways. This aligns with the predicted functional reprogramming pattern, rather than uniform upregulation or downregulation across the entire pathway (Fig. 6; Table S8).

Among LUAD-downregulated subsets, module scores were significantly decreased for biofilm/QS, peptidoglycan-related functions, beta-lactam-associated functions, secretion systems, tryptophan/indole metabolism, and nitrogen/sulfur metabolism (FDR < 0.05; Fig. 6). Conversely, within LUAD-upregulated subsets, early-stage LUAD tissues showed significant increases for nitrogen/sulfur metabolism, beta-lactam-associated functions, tryptophan/indole metabolism, and peptidoglycan-related functions (FDR < 0.05; Fig. 6). Collectively, these patterns suggest that early-stage LUAD may be associated with a redistribution of predicted functional potentials spanning microbial cell-envelope programs (peptidoglycan-related functions), ecological and interaction traits (biofilm/QS and secretion systems), and immunometabolic axes (tryptophan/indole and nitrogen/sulfur metabolism).

## DISCUSSION

Commensal microbes in the human body have attracted increasing interest as potential modulators and therapeutic targets in cancer (35). Although lung tumors harbor relatively low microbial biomass, accumulating evidence links tumor-associated microbial composition to lung cancer progression (36). However, studies of tissue microbiota remain technically challenging, as results can be strongly influenced by sampling location, sequencing depth, and background contamination. In this study, we analyzed the microbial features of early-stage LUAD tissues and partially matched noncancerous tissues using an ASV-based pipeline with contamination modeling and paired-design statistical analyses. Our findings may inform future efforts to develop microbial biomarkers for early detection and to guide subsequent therapeutic stratification, while recognizing that further validation in independent cohorts will be required.

A key methodological strength of this study is the use of patient-aware analyses for low-biomass lung tissues. Because quality control yielded a partially paired design, within-patient correlation was modeled using LMM with patient ID as a random effect, rather than treating samples as fully independent or restricting inference to complete pairs, which remains common in prior LUAD tissue microbiome reports (17, 20, 21, 37, 38). In addition, marker discovery relied on patient-level resampling with aggregation of out-of-bag predictions. This approach reduces the risk of information leakage between matched samples and provides a more conservative estimate of discriminative performance than apparent training metrics.

When comparing the microbiota in early-stage LUAD tissues with NC tissues, we observed that early-stage LUAD tissues exhibited an expansion of low-prevalence taxa rather than a broad shift in community evenness, which is consistent with previous findings (39). Simultaneously, β-diversity analysis revealed that differences between the two groups were more pronounced at presence/absence-based (unweighted) metrics than with abundance-weighted metrics. This suggests that early-stage LUAD tissues exhibit an expansion of low-prevalence microbes compared to NC tissues, whereas the dominant community structure remains largely unchanged. Similar patterns have been observed across diverse disease settings in which local ecological constraints and host regulation are perturbed. In such settings, low-prevalence members may become enriched without a wholesale shift in dominant taxa (40, 41). Reported changes in lung tissue microbial α-diversity in lung cancer are inconsistent (42–44), potentially reflecting differences in geography, specimen type, lifestyle factors, medication exposure, and low-biomass-related technical variability.

We observed taxonomic differences between early-stage LUAD tissues and NC tissues. *Pseudomonadota*, *Bacteroidota*, *Bacillota*, and *Actinobacteriota* dominated both groups, consistent with prior lung tissue microbiome profiles (45). Differences became more apparent at finer taxonomic resolution. At the family and genus levels, both groups were characterized by a limited set of high-abundance taxa, whereas early-stage LUAD tissues additionally harbored a larger number of low-prevalence members. *Achromobacter*, *Pedobacter*, and *Pseudomonas* collectively constituted approximately half of the total community in both early-stage LUAD and NC tissues. *Achromobacter* showed a non-significant reduction in tumors and has been reported in airway infection contexts (46, 47). *Pedobacter* has been infrequently linked to human disease, but some studies indicate reduced abundance in the gut of colon cancer patients (48, 49). In contrast, *Pseudomonas* has been consistently identified as a core lung microbe, present in both healthy individuals and lung cancer patients (5, 50–52). Notably, *Alcanivoracaceae* and *Ketobacter* were detected only in early-stage LUAD tissues in the present cohort. Because these microbes were described as environmental taxa with metabolic capacities related to hydrocarbon substrates and microplastics, one possible interpretation is that tumor environments may provide conditions that facilitate their persistence or detection (53–55). Nevertheless, a causal relationship with LUAD cannot be inferred from the present data.

Differential abundance testing that accounted for patient pairing identified a relatively small set of genera associated with early-stage LUAD. Two taxa were significantly enriched in tumors, including *unclassified_f__Oscillospiraceae* and *norank_c__Eremiobacteria*. In contrast, *Aquabacterium*, *Sphingobium*, *Roseateles*, *Jeotgalibaca*, and *unclassified_f__Devosiaceae* were significantly decreased in early-stage LUAD. These findings align with observations from diversity analyses, indicating that the differences in early-stage LUAD are primarily driven by low-prevalence taxa rather than uniform changes in dominant members. Meanwhile, *Romboutsia* and *Bacillus* showed a directionally consistent increase in LUAD with near-threshold associations (prevalence > 0.4). The complementary results of LEfSe highlighted discriminant clades consistent with these trends, including lineages within *Bacilli* and *Gammaproteobacteria*. Collectively, these results suggest that early-stage LUAD is associated with selective change in a small subset of taxa superimposed on an otherwise conserved microbial community backbone.

We also constructed a biomarker panel through patient-level resampling and stability selection. Cluster bootstrap resampling at the patient level, together with aggregation of out-of-bag predictions, provides a more conservative estimate of discriminative performance than apparent training AUC and reduces the risk of information leakage (56). Notably, the stable microbes were not restricted to tumor-enriched taxa. Instead, the panel reflected an imbalance characterized by retention of a tumor-associated genus (*Bacillus*), alongside reduced abundance of several genera more prominent in NC tissues, including *Staphylococcus*, which is frequently detected in respiratory microbiota (5, 57, 58). This pattern is consistent with dysbiosis in which early-stage LUAD gains additional low-prevalence members while certain resident taxa decrease.

An additional consideration is whether lung cancer microbiota change with disease stage. Prior studies have reported stage-related microbial patterns in cancer tissues and airway samples (42, 59–61). In a large lung tissue study, *Thermus* was reported to be more abundant in advanced-stage (IIIB/IV) patients, whereas *Legionella* was associated with patients who developed metastases (42). Beyond individual taxa, a LUAD microbiota study found that microbial co-abundance networks increase in topological complexity with advancing stage (60). Taken together, these findings support that microbiota composition and community organization can vary across stages of lung cancer. Biologically, the progression of LUAD from early to advanced stages may induce more intense and heterogeneous ecological pressures, such as alterations in airway patency, hypoxia and necrosis, immune remodeling, and therapeutic exposure (62, 63). These factors may further reshape community membership and correlation structure. In this context, focusing on early-stage or untreated LUAD may better capture microbiota alterations while minimizing the extensive interference caused by intensive treatments and advanced systemic effects (17, 64). However, since this cohort primarily comprises early-stage LUAD, the data are not suited to defining stage-dependent disease progression. To validate whether the taxa and biomarker signatures identified in early-stage LUAD persist, diminish, or are replaced in later stages will require cohorts spanning broader stage distributions. Nevertheless, establishing reproducible early-stage signatures remains clinically significant, as this time window is most directly relevant to the development of biomarkers for early detection and risk stratification.

Correlation network analysis indicated higher connectivity and local clustering in early-stage LUAD tissues than in NC tissues, together with a shift in highly central taxa. Because networks were inferred from Spearman correlations on compositional abundance profiles, these patterns should be interpreted as co-variation structure rather than direct ecological interactions or evidence of community stability (65, 66). The denser and more locally clustered correlation structure in early-stage LUAD tissues may reflect stronger shared ecological constraints in the tumor microenvironment, whereby subsets of taxa co-vary under common external drivers (67). At the same time, correlation networks inferred from low-biomass, sparse communities can be sensitive to low-prevalence features and thresholding, which may affect edge recovery and inflate apparent connectivity (68). Together, these considerations suggest that the observed network tightening is consistent with tumor-associated ecological filtering, while remaining cautious that part of the signal could be influenced by sparsity-related network reconstruction effects. Notably, our bootstrap-based stability assessment indicates that the main topological differences were not driven by a small number of unstable edges or samples. It remains to be validated whether these microbial network changes synchronize with shifts in community stability through cross-cohort and quantitative robustness analyses.

Studies have reported inconsistent pathway-level effects based on 16S-based functional inference in early-stage lung cancer (20, 69, 70). While we found no significant differences at the pathway level, KO-level analysis revealed a within-module redistribution. Specifically, within the same module, distinct KO subsets shifted in opposite directions across the cell-envelope, biofilm/QS, tryptophan/indole, and nitrogen/sulfur modules. Prior studies and reviews have frequently implicated host-microbe interface functions in lung cancer tissues, including reported alterations in LPS programs (69, 71). In our data set, in addition to cell-envelope host-microbe interface, tryptophan/indole remodeling represents an intriguing observation, as the microbial indole metabolite indole-3-acetic acid influences antitumor immunity (72, 73). Nevertheless, these results represent inferred functional potential and warrant confirmation using shotgun metagenomics and targeted metabolite measurements.

Our study has some limitations. First, despite contamination modeling and negative controls, lung tissue microbiota analysis remains susceptible to background signals, and independent validation is essential. Second, 16S rRNA sequencing provides limited taxonomic resolution and cannot directly establish microbial activity, so PICRUSt2 predictions should be validated using shotgun metagenomics. Third, the present cohort focuses on early-stage disease; extending analyses to stage-spanning cohorts and additional clinical phenotypes will be required to determine whether microbial patterns vary with progression. Finally, the sample size limited the ability to examine interactions between microbiota and clinical covariates. Despite these limitations, the paired design, stability-driven biomarker selection, and conservative internal validation collectively support the presence of reproducible microbiota alterations in early-stage LUAD that warrant further mechanistic and translational investigation.

### Conclusion

We utilized 16S rRNA sequencing to investigate the composition and structure of the microbiota in early-stage LUAD tissues compared to noncancerous lung tissues. Early-stage LUAD was associated with the expansion of low-prevalence taxa and shifts in community composition, without a global change in evenness. We identified a set of early-stage LUAD-associated genera and obtained microbial features with discriminatory potential through stability screening. Correlation network analysis further indicated a denser and more locally clustered co-occurrence structure in LUAD tissues, accompanied by a shift in central taxa. Functional inference based on 16S rRNA sequencing further suggests a redistribution of microbial functional potential across selected host-microbe interaction modules. Our findings provide a framework for developing lung microbiota-based biomarkers for early-stage LUAD, thereby contributing to a better understanding of the role of lung microbiota in the progression of lung cancer.

## Data Availability

Raw 16S rRNA sequencing data were uploaded to the National Center for Biotechnology Information (NCBI) Sequence Read Archive database (accession number: PRJNA1220209).

## SUPPLEMENTAL MATERIAL

Table S1 spectrum.01987-25-s0001.xlsx Per-sample sequencing depth and quality-control statistics for lung tissue 16S rRNA datasets, including read retention after denoising and chimera filtering.

Table S2 spectrum.01987-25-s0002.xlsx Genus-level differential abundance results comparing NC and early-stage LUAD tissues using MaAsLin2.

Table S3 spectrum.01987-25-s0003.xlsx Patient-level cluster-bootstrap stability selection results for genus-level markers, along with Elastic Net model coefficients for the selected diagnostic signature.

Table S4 spectrum.01987-25-s0004.xlsx Discriminative performance of the diagnostic model in apparent training fit and pooled OOB validation.

Table S5 spectrum.01987-25-s0005.xlsx Centrality metrics of taxa in the NC correlation network, summarizing node-level degree, closeness, and betweenness.

Table S6 spectrum.01987-25-s0006.xlsx Centrality metrics of taxa in the early-stage LUAD correlation network, summarizing node-level degree, closeness, and betweenness.

Table S7 spectrum.01987-25-s0007.xlsx Patient-level bootstrap stability analysis of correlation networks and identification of robust core nodes in NC and early-stage LUAD tissues.

Table S8 spectrum.01987-25-s0008.xlsx PICRUSt2-inferred functional associations between NC and early-stage LUAD tissues at KEGG Level 3 pathway, KO, and module levels, including multiple-testing results.

ASM does not own the copyrights to Supplemental Material that may be linked to, or accessed through, an article. The authors have granted ASM a non-exclusive, world-wide license to publish the Supplemental Material files. Please contact the corresponding author directly for reuse.

## References

[1] Miller KD, Fidler-Benaoudia M, Keegan TH, Hipp HS, Jemal A, Siegel RL. 2020. Cancer statistics for adolescents and young adults, 2020. CA Cancer J Clin 70:443–459. doi:10.3322/caac.21637 32940362

[2] Sung H, Ferlay J, Siegel RL, Laversanne M, Soerjomataram I, Jemal A, Bray F. 2021. Global Cancer Statistics 2020: GLOBOCAN estimates of incidence and mortality worldwide for 36 cancers in 185 countries. CA Cancer J Clin 71:209–249. doi:10.3322/caac.21660 33538338

[3] Shende P, Augustine S, Prabhakar B, Gaud RS. 2019. Advanced multimodal diagnostic approaches for detection of lung cancer. Expert Rev Mol Diagn 19:409–417. doi:10.1080/14737159.2019.1607299 30977684

[4] Zeng W, Zhao C, Yu M, Chen H, Pan Y, Wang Y, Bao H, Ma H, Ma S. 2022. Alterations of lung microbiota in patients with non-small cell lung cancer. Bioengineered 13:6665–6677. doi:10.1080/21655979.2022.2045843 35254206 PMC8973753

[5] Gomes S, Cavadas B, Ferreira JC, Marques PI, Monteiro C, Sucena M, Sousa C, Vaz Rodrigues L, Teixeira G, Pinto P, Tavares de Abreu T, Bárbara C, Semedo J, Mota L, Carvalho AS, Matthiesen R, Pereira L, Seixas S. 2019. Profiling of lung microbiota discloses differences in adenocarcinoma and squamous cell carcinoma. Sci Rep 9:12838. doi:10.1038/s41598-019-49195-w 31492894 PMC6731246

[6] Hilty M, Burke C, Pedro H, Cardenas P, Bush A, Bossley C, Davies J, Ervine A, Poulter L, Pachter L, Moffatt MF, Cookson WOC. 2010. Disordered microbial communities in asthmatic airways. PLoS One 5:e8578. doi:10.1371/journal.pone.0008578 20052417 PMC2798952

[7] Charlson ES, Bittinger K, Haas AR, Fitzgerald AS, Frank I, Yadav A, Bushman FD, Collman RG. 2011. Topographical continuity of bacterial populations in the healthy human respiratory tract. Am J Respir Crit Care Med 184:957–963. doi:10.1164/rccm.201104-0655OC 21680950 PMC3208663

[8] Erb-Downward JR, Thompson DL, Han MK, Freeman CM, McCloskey L, Schmidt LA, Young VB, Toews GB, Curtis JL, Sundaram B, Martinez FJ, Huffnagle GB. 2011. Analysis of the lung microbiome in the “healthy” smoker and in COPD. PLoS One 6:e16384. doi:10.1371/journal.pone.0016384 21364979 PMC3043049

[9] Zhao Q, Wu J, Ding Y, Pang Y, Jiang C. 2023. Gut microbiota, immunity, and bile acid metabolism: decoding metabolic disease interactions. Life Metab 2:load032. doi:10.1093/lifemeta/load032 39872860 PMC11749371

[10] Man WH, de Steenhuijsen Piters WAA, Bogaert D. 2017. The microbiota of the respiratory tract: gatekeeper to respiratory health. Nat Rev Microbiol 15:259–270. doi:10.1038/nrmicro.2017.14 28316330 PMC7097736

[11] Gokulan K, Joshi M, Khare S, Bartter T. 2022. Lung microbiome, gut-lung axis and chronic obstructive pulmonary disease. Curr Opin Pulm Med 28:134–138. doi:10.1097/MCP.0000000000000853 34907959

[12] Sethi S, Maloney J, Grove L, Wrona C, Berenson CS. 2006. Airway inflammation and bronchial bacterial colonization in chronic obstructive pulmonary disease. Am J Respir Crit Care Med 173:991–998. doi:10.1164/rccm.200509-1525OC 16474030 PMC2662918

[13] Yang D, Chen X, Wang J, Lou Q, Lou Y, Li L, Wang H, Chen J, Wu M, Song X, Qian Y. 2019. Dysregulated lung commensal bacteria drive interleukin-17B production to promote pulmonary fibrosis through their outer membrane vesicles. Immunity 50:692–706. doi:10.1016/j.immuni.2019.02.001 30824326

[14] Tsay J-CJ, Wu BG, Badri MH, Clemente JC, Shen N, Meyn P, Li Y, Yie T-A, Lhakhang T, Olsen E, Murthy V, Michaud G, Sulaiman I, Tsirigos A, Heguy A, Pass H, Weiden MD, Rom WN, Sterman DH, Bonneau R, Blaser MJ, Segal LN. 2018. Airway microbiota is associated with upregulation of the PI3K pathway in lung cancer. Am J Respir Crit Care Med 198:1188–1198. doi:10.1164/rccm.201710-2118OC 29864375 PMC6221574

[15] Jin C, Lagoudas GK, Zhao C, Bullman S, Bhutkar A, Hu B, Ameh S, Sandel D, Liang XS, Mazzilli S, Whary MT, Meyerson M, Germain R, Blainey PC, Fox JG, Jacks T. 2019. Commensal microbiota promote lung cancer development via γδ T cells. Cell 176:998–1013. doi:10.1016/j.cell.2018.12.040 30712876 PMC6691977

[16] Wong LM, Shende N, Li WT, Castaneda G, Apostol L, Chang EY, Ongkeko WM. 2020. Comparative analysis of age- and gender-associated microbiome in lung adenocarcinoma and lung squamous cell carcinoma. Cancers (Basel) 12:1447. doi:10.3390/cancers12061447 32498338 PMC7352186

[17] Ma Y, Qiu M, Wang S, Meng S, Yang F, Jiang G. 2021. Distinct tumor bacterial microbiome in lung adenocarcinomas manifested as radiological subsolid nodules. Transl Oncol 14:101050. doi:10.1016/j.tranon.2021.101050 33765542 PMC8022255

[18] Shouhua Z, Meilan L. 2023. Microbial diversity and abundance in pulmonary tissue of patients with early-stage lung cancer. Jundishapur J Microbiol 16. doi:10.5812/jjm-137478

[19] Yiminniyaze R, Zhang Y, Zhu N, Zhang X, Wang J, Li C, Wumaier G, Zhou D, Xia J, Li S, Dong L, Zhang Y, Zhang Y, Li S. 2025. Characterizations of lung cancer microbiome and exploration of potential microbial risk factors for lung cancer. Sci Rep 15:15683. doi:10.1038/s41598-025-98424-y 40325116 PMC12052825

[20] Su K, Gao Y, He J. 2023. A comparison of the microbiome composition in lower respiratory tract at different sites in early lung cancer patients. Transl Lung Cancer Res 12:1264–1275. doi:10.21037/tlcr-23-231 37425420 PMC10326795

[21] Sun Y, Gan Z, Wang X, Liu J, Zhong W, Zhang Z, Zuo J, Zhong H, Huang X, Yan Z, Cao Q. 2024. Integrative metagenomic, transcriptomic, and proteomic analysis reveal the microbiota-host interplay in early-stage lung adenocarcinoma among non-smokers. J Transl Med 22:652. doi:10.1186/s12967-024-05485-0 38997719 PMC11245786

[22] Liu J, Zhang M, Zhang R, Zhu W, Mao S. 2016. Comparative studies of the composition of bacterial microbiota associated with the ruminal content, ruminal epithelium and in the faeces of lactating dairy cows. Microb Biotechnol 9:257–268. doi:10.1111/1751-7915.12345 26833450 PMC4767291

[23] Austin GI, Park H, Meydan Y, Seeram D, Sezin T, Lou YC, Firek BA, Morowitz MJ, Banfield JF, Christiano AM, Pe’er I, Uhlemann AC, Shenhav L, Korem T. 2023. Contamination source modeling with SCRuB improves cancer phenotype prediction from microbiome data. Nat Biotechnol 41:1820–1828. doi:10.1038/s41587-023-01696-w 36928429 PMC10504420

[24] Zhang S, Lv K, Liu Z, Zhao R, Li F. 2024. Fatty acid metabolism of immune cells: a new target of tumour immunotherapy. Cell Death Discov 10:39. doi:10.1038/s41420-024-01807-9 38245525 PMC10799907

[25] Wang Y, Miao Z, Qin X, Li B, Han Y. 2021. NOD2 deficiency confers a pro-tumorigenic macrophage phenotype to promote lung adenocarcinoma progression. J Cellular Molecular Medi 25:7545–7558. doi:10.1111/jcmm.16790 PMC8335701 34268854

[26] Rutkowski MR, Stephen TL, Svoronos N, Allegrezza MJ, Tesone AJ, Perales-Puchalt A, Brencicova E, Escovar-Fadul X, Nguyen JM, Cadungog MG, Zhang R, Salatino M, Tchou J, Rabinovich GA, Conejo-Garcia JR. 2015. Microbially driven TLR5-dependent signaling governs distal malignant progression through tumor-promoting inflammation. Cancer Cell 27:27–40. doi:10.1016/j.ccell.2014.11.009 25533336 PMC4293269

[27] Cao Y, Xia H, Tan X, Shi C, Ma Y, Meng D, Zhou M, Lv Z, Wang S, Jin Y. 2024. Intratumoural microbiota: a new frontier in cancer development and therapy. Signal Transduct Target Ther 9:15. doi:10.1038/s41392-023-01693-0 38195689 PMC10776793

[28] Choi E, Murray B, Choi S. 2023. Biofilm and cancer: interactions and future directions for cancer therapy. Int J Mol Sci 24:12836. doi:10.3390/ijms241612836 37629016 PMC10454087

[29] Geller LT, Barzily-Rokni M, Danino T, Jonas OH, Shental N, Nejman D, Gavert N, Zwang Y, Cooper ZA, Shee K, et al.. 2017. Potential role of intratumor bacteria in mediating tumor resistance to the chemotherapeutic drug gemcitabine. Science 357:1156–1160. doi:10.1126/science.aah5043 28912244 PMC5727343

[30] Luu M, Riester Z, Baldrich A, Reichardt N, Yuille S, Busetti A, Klein M, Wempe A, Leister H, Raifer H, et al.. 2021. Microbial short-chain fatty acids modulate CD8^+^ T cell responses and improve adoptive immunotherapy for cancer. Nat Commun 12:4077. doi:10.1038/s41467-021-24331-1 34210970 PMC8249424

[31] Bender MJ, McPherson AC, Phelps CM, Pandey SP, Laughlin CR, Shapira JH, Medina Sanchez L, Rana M, Richie TG, Mims TS, Gocher-Demske AM, Cervantes-Barragan L, Mullett SJ, Gelhaus SL, Bruno TC, Cannon N, McCulloch JA, Vignali DAA, Hinterleitner R, Joglekar AV, Pierre JF, Lee STM, Davar D, Zarour HM, Meisel M. 2023. Dietary tryptophan metabolite released by intratumoral Lactobacillus reuteri facilitates immune checkpoint inhibitor treatment. Cell 186:1846–1862. doi:10.1016/j.cell.2023.03.011 37028428 PMC10148916

[32] Perez-Castro L, Garcia R, Venkateswaran N, Barnes S, Conacci-Sorrell M. 2023. Tryptophan and its metabolites in normal physiology and cancer etiology. FEBS J 290:7–27. doi:10.1111/febs.16245 34687129 PMC9883803

[33] Kurmi K, Haigis MC. 2020. Nitrogen metabolism in cancer and immunity. Trends Cell Biol 30:408–424. doi:10.1016/j.tcb.2020.02.005 32302552 PMC7386658

[34] Moon JY, Kye BH, Ko SH, Yoo RN. 2023. Sulfur metabolism of the gut microbiome and colorectal cancer: the threat to the younger generation. Nutrients 15:1966. doi:10.3390/nu15081966 37111185 PMC10146533

[35] Ting NL-N, Lau HC-H, Yu J. 2022. Cancer pharmacomicrobiomics: targeting microbiota to optimise cancer therapy outcomes. Gut 71:1412–1425. doi:10.1136/gutjnl-2021-326264 35277453 PMC9185832

[36] Sun Y, Wen M, Liu Y, Wang Y, Jing P, Gu Z, Jiang T, Wang W. 2023. The human microbiome: a promising target for lung cancer treatment. Front Immunol 14:1091165. doi:10.3389/fimmu.2023.1091165 36817461 PMC9936316

[37] Yang K, Wang S, Ding Z, Zhang K, Zhu W, Wang H, Pan M, Li X, Wang H, Yu Z. 2024. Unveiling microbial dynamics in lung adenocarcinoma and adjacent nontumor tissues: insights from nicotine exposure and diverse clinical stages via nanopore sequencing technology. Front Cell Infect Microbiol 14:1397989. doi:10.3389/fcimb.2024.1397989 39258251 PMC11385298

[38] Zhou Y, Zeng H, Liu K, Pan H, Wang B, Zhu M, Wang J, Wang H, Chen H, Shen D, Wang Y, Yu Z. 2023. Microbiota profiles in the saliva, cancerous tissues and its companion paracancerous tissues among Chinese patients with lung cancer. BMC Microbiol 23:237. doi:10.1186/s12866-023-02882-1 37641037 PMC10464170

[39] Wang J, Su W, Chen Q, Zhou J, Wang X, Jiang R, Li J, Xing P. 2025. Microbiome-metabolome dysbiosis of bronchoalveolar lavage fluid of lung cancer patients. Front Microbiol 16:1669172. doi:10.3389/fmicb.2025.1669172 41311499 PMC12647101

[40] Helmink BA, Khan MAW, Hermann A, Gopalakrishnan V, Wargo JA. 2019. The microbiome, cancer, and cancer therapy. Nat Med 25:377–388. doi:10.1038/s41591-019-0377-7 30842679

[41] Zhang W, Xiang Y, Ren H, Liu Y, Wang Q, Ran M, Zhou W, Tian L, Zheng X, Qiao C, Liu Y, Yan M. 2025. The tumor microbiome in cancer progression: mechanisms and therapeutic potential. Mol Cancer 24:195. doi:10.1186/s12943-025-02403-w 40665305 PMC12261620

[42] Kim OH, Choi BY, Kim DK, Kim NH, Rho JK, Sul WJ, Lee SW. 2022. The microbiome of lung cancer tissue and its association with pathological and clinical parameters. Am J Cancer Res 12:2350–2362. 35693079 PMC9185621

[43] Apopa PL, Alley L, Penney RB, Arnaoutakis K, Steliga MA, Jeffus S, Bircan E, Gopalan B, Jin J, Patumcharoenpol P, Jenjaroenpun P, Wongsurawat T, Shah N, Boysen G, Ussery D, Nookaew I, Fagan P, Bebek G, Orloff MS. 2018. PARP1 is up-regulated in non-small cell lung cancer tissues in the presence of the cyanobacterial toxin microcystin. Front Microbiol 9:1757. doi:10.3389/fmicb.2018.01757 30127774 PMC6087756

[44] Patnaik SK, Cortes EG, Kannisto ED, Punnanitinont A, Dhillon SS, Liu S, Yendamuri S. 2021. Lower airway bacterial microbiome may influence recurrence after resection of early-stage non-small cell lung cancer. J Thorac Cardiovasc Surg 161:419–429. doi:10.1016/j.jtcvs.2020.01.104 32340803

[45] Greathouse KL, White JR, Vargas AJ, Bliskovsky VV, Beck JA, von Muhlinen N, Polley EC, Bowman ED, Khan MA, Robles AI, et al.. 2018. Interaction between the microbiome and TP53 in human lung cancer. Genome Biol 19:123. doi:10.1186/s13059-018-1501-6 30143034 PMC6109311

[46] Chandrasekar PH, Arathoon E, Levine DP. 1986. Infections due to Achromobacter xylosoxidans. Case report and review of the literature. Infection 14:279–282. doi:10.1007/BF01643962 3818105

[47] Swenson CE, Sadikot RT. 2015. Achromobacter respiratory infections. Ann Am Thorac Soc 12:252–258. doi:10.1513/AnnalsATS.201406-288FR 25706494

[48] Gao Z, Guo B, Gao R, Zhu Q, Qin H. 2015. Microbiota disbiosis is associated with colorectal cancer. Front Microbiol 6:20. doi:10.3389/fmicb.2015.00020 25699023 PMC4313696

[49] Leung PHM, Subramanya R, Mou Q, Lee KT-W, Islam F, Gopalan V, Lu C-T, Lam AK-Y. 2019. Characterization of mucosa-associated microbiota in matched cancer and non-neoplastic mucosa from patients with colorectal cancer. Front Microbiol 10:1317. doi:10.3389/fmicb.2019.01317 31244818 PMC6581718

[50] Liu HX, Tao LL, Zhang J, Zhu YG, Zheng Y, Liu D, Zhou M, Ke H, Shi MM, Qu JM. 2018. Difference of lower airway microbiome in bilateral protected specimen brush between lung cancer patients with unilateral lobar masses and control subjects. Int J Cancer 142:769–778. doi:10.1002/ijc.31098 29023689

[51] Sun Y, Liu Y, Li J, Tan Y, An T, Zhuo M, Pan Z, Ma M, Jia B, Zhang H, Wang Z, Yang R, Bi Y. 2023. Characterization of lung and oral microbiomes in lung cancer patients using culturomics and 16S rRNA gene sequencing. Microbiol Spectr 11:e0031423. doi:10.1128/spectrum.00314-23 37092999 PMC10269771

[52] Koslow M, Epstein Shochet G, Matveychuk A, Israeli-Shani L, Guber A, Shitrit D. 2017. The role of bacterial culture by bronchoscopy in patients with lung cancer: a prospective study. J Thorac Dis 9:5300–5305. doi:10.21037/jtd.2017.10.150 29312739 PMC5756955

[53] Singleton SL, Davis EW II, Arnold HK, Daniels AMY, Brander SM, Parsons RJ, Sharpton TJ, Giovannoni SJ. 2023. Identification of rare microbial colonizers of plastic materials incubated in a coral reef environment. Front Microbiol 14:1259014. doi:10.3389/fmicb.2023.1259014 37869676 PMC10585116

[54] Chen Z, Sun W, Wang S, Yang J, Huang W, Huang D, Jiang K, Zhang X, Sun X. 2024. Interactions between microplastics and organic contaminants: the microbial mechanisms for priming effects of organic compounds on microplastic biodegradation. Water Res 267:122523. doi:10.1016/j.watres.2024.122523 39353345

[55] Kim SH, Kim JG, Jung MY, Kim SJ, Gwak JH, Yu WJ, Roh SW, Kim YH, Rhee SK. 2018. Ketobacter alkanivorans gen. nov., sp. nov., an n-alkane-degrading bacterium isolated from seawater. Int J Syst Evol Microbiol 68:2258–2264. doi:10.1099/ijsem.0.002823 29809120

[56] Bouwmeester W, Moons KGM, Kappen TH, van Klei WA, Twisk JWR, Eijkemans MJC, Vergouwe Y. 2013. Internal validation of risk models in clustered data: a comparison of bootstrap schemes. Am J Epidemiol 177:1209–1217. doi:10.1093/aje/kws396 23660796

[57] Lipinksi JH, Ranjan P, Dickson RP, O’Dwyer DN. 2024. The lung microbiome. J Immunol 212:1269–1275. doi:10.4049/jimmunol.2300716 38560811 PMC11073614

[58] Baranova E, Druzhinin V, Matskova L, Demenkov P, Volobaev V, Minina V, Larionov A, Titov V. 2022. Sputum microbiome composition in patients with squamous cell lung carcinoma. Life (Basel) 12:1365. doi:10.3390/life12091365 36143401 PMC9501211

[59] Yu G, Gail MH, Consonni D, Carugno M, Humphrys M, Pesatori AC, Caporaso NE, Goedert JJ, Ravel J, Landi MT. 2016. Characterizing human lung tissue microbiota and its relationship to epidemiological and clinical features. Genome Biol 17:163. doi:10.1186/s13059-016-1021-1 27468850 PMC4964003

[60] Su Y, Li S, Sang D, Zhang Y. 2024. The characteristics of intratumoral microbial community reflect the development of lung adenocarcinoma. Front Microbiol 15:1353940. doi:10.3389/fmicb.2024.1353940 38721596 PMC11076736

[61] Huang DH, He J, Su XF, Wen YN, Zhang SJ, Liu LY, Zhao H, Ye CP, Wu JH, Cai S, Dong H. 2022. The airway microbiota of non-small cell lung cancer patients and its relationship to tumor stage and EGFR gene mutation. Thorac Cancer 13:858–869. doi:10.1111/1759-7714.14340 35142041 PMC8930493

[62] Li R, Li J, Zhou X. 2024. Lung microbiome: new insights into the pathogenesis of respiratory diseases. Signal Transduct Target Ther 9:19. doi:10.1038/s41392-023-01722-y 38228603 PMC10791971

[63] El Tekle G, Garrett WS. 2023. Bacteria in cancer initiation, promotion and progression. Nat Rev Cancer 23:600–618. doi:10.1038/s41568-023-00594-2 37400581

[64] Elkrief A, Méndez-Salazar EO, Maillou J, Vanderbilt CM, Gogia P, Desilets A, Messaoudene M, Kelly D, Ladanyi M, Hellmann MD, Zitvogel L, Rudin CM, Routy B, Derosa L, Schoenfeld AJ. 2024. Antibiotics are associated with worse outcomes in lung cancer patients treated with chemotherapy and immunotherapy. NPJ Precis Oncol 8:143. doi:10.1038/s41698-024-00630-w 39014160 PMC11252311

[65] Röttjers L, Faust K. 2018. From hairballs to hypotheses-biological insights from microbial networks. FEMS Microbiol Rev 42:761–780. doi:10.1093/femsre/fuy030 30085090 PMC6199531

[66] Poudel R, Jumpponen A, Schlatter DC, Paulitz TC, Gardener BBM, Kinkel LL, Garrett KA. 2016. Microbiome networks: a systems framework for identifying candidate microbial assemblages for disease management. Phytopathology 106:1083–1096. doi:10.1094/PHYTO-02-16-0058-FI 27482625

[67] Faust K, Raes J. 2012. Microbial interactions: from networks to models. Nat Rev Microbiol 10:538–550. doi:10.1038/nrmicro2832 22796884

[68] Pust M-M, Tümmler B. 2022. Bacterial low-abundant taxa are key determinants of a healthy airway metagenome in the early years of human life. Comput Struct Biotechnol J 20:175–186. doi:10.1016/j.csbj.2021.12.008 35024091 PMC8713036

[69] Han W, Wang N, Han M, Liu X, Sun T, Xu J. 2023. Identification of microbial markers associated with lung cancer based on multi-cohort 16 s rRNA analyses: a systematic review and meta-analysis. Cancer Med 12:19301–19319. doi:10.1002/cam4.6503 37676050 PMC10557844

[70] Peters BA, Pass HI, Burk RD, Xue X, Goparaju C, Sollecito CC, Grassi E, Segal LN, Tsay J-CJ, Hayes RB, Ahn J. 2022. The lung microbiome, peripheral gene expression, and recurrence-free survival after resection of stage II non-small cell lung cancer. Genome Med 14:121. doi:10.1186/s13073-022-01126-7 36303210 PMC9609265

[71] Bou Zerdan M, Kassab J, Meouchy P, Haroun E, Nehme R, Bou Zerdan M, Fahed G, Petrosino M, Dutta D, Graziano S. 2022. The lung microbiota and lung cancer: a growing relationship. Cancers (Basel) 14:4813. doi:10.3390/cancers14194813 36230736 PMC9563611

[72] Tintelnot J, Xu Y, Lesker TR, Schönlein M, Konczalla L, Giannou AD, Pelczar P, Kylies D, Puelles VG, Bielecka AA, et al.. 2023. Microbiota-derived 3-IAA influences chemotherapy efficacy in pancreatic cancer. Nature 615:168–174. doi:10.1038/s41586-023-05728-y 36813961 PMC9977685

[73] Hezaveh K, Shinde RS, Klötgen A, Halaby MJ, Lamorte S, Ciudad MT, Quevedo R, Neufeld L, Liu ZQ, Jin R, et al.. 2022. Tryptophan-derived microbial metabolites activate the aryl hydrocarbon receptor in tumor-associated macrophages to suppress anti-tumor immunity. Immunity 55:324–340. doi:10.1016/j.immuni.2022.01.006 35139353 PMC8888129

